# Genetic epidemiology approach to estimating birth incidence and current disease prevalence for rhizomelic chondrodysplasia punctata

**DOI:** 10.1186/s13023-021-01889-z

**Published:** 2021-07-06

**Authors:** Tarik Luisman, Tara Smith, Shawn Ritchie, Karen E. Malone

**Affiliations:** 1 Leiden Analytics, Leiden, The Netherlands; 2 Med-Life Discoveries, Saskatoon, SK Canada

**Keywords:** Epidemiology, Rare disease, Plasmalogen, PEX7, GNPAT, AGPS, FAR1, PEX5, Rhizomelic chondrodysplasia punctate

## Abstract

**Background:**

Rhizomelic chondrodysplasia punctata (RCDP) is an inherited ultra-rare disease which results in severely impaired physical and mental development. Mutations in one of five genes involved in plasmalogen biosynthesis have been reported to drive disease pathology. Estimates of disease incidence have been extremely challenging due to the rarity of the disorder, preventing an understanding of the unmet medical need. To address this, we have prepared a disease incidence and prevalence model based on genetic epidemiology approaches to estimate the total number of RCDP patients affected, and their demographic characteristics.

**Results:**

Extraction of allelic frequencies for known and predicted pathogenic variants in *PEX7, GNPAT, AGPS, FAR1, PEX5* (limited to the PTS2 domain encoding region) genes, from large-scale human genetic diversity datasets (TopMed and gnomAD) revealed the mutational landscape contributing to the RCDP patient population in the US and Europe. We computed genetic prevalence to derive birth incidence for RCDP and modeled the impact to life expectancy to obtain high confidence estimates of disease prevalence. Our population genetics-based model indicates PEX7 variants are expected to contribute to the majority of RCDP cases in both the US and Europe; closely aligning with clinical reports. Furthermore, this model provides estimates for RCDP subtypes due to mutations in other genes, including exceedingly rare subtypes.

**Conclusion:**

In total, the estimated number of RCDP patients in the US and the five largest European countries (UK, Germany, France, Italy and Spain) is between 516 and 847 patients, all under the age of 35 years old. This model provides a quantitative framework for better understanding the unmet medical need in RCDP, to help guide disease awareness and diagnosis efforts for this specific patient group.

**Supplementary Information:**

The online version contains supplementary material available at 10.1186/s13023-021-01889-z.

## Background

Rhizomelic chondrodysplasia punctata (RCDP) is an ultra-rare inherited disorder caused by an impaired ability to synthesize plasmalogens. Plasmalogens are vinyl-ether containing membrane phospholipids that play a critical role in maintaining the proper structure and function of the cellular membrane. There is a range of disease presentations, however the hallmark characteristics are skeletal dysplasia, congenital cataracts, and profound growth and developmental delays. Skeletal dysplasia involves the proximal shortening of the long bones (rhizomelia) and abnormal mineralization of the growth plates (chondrodysplasia punctata), resulting in limited joint mobility. Dramatically reduced life expectancy is common with RCDP patients, with survival varying widely with the severity of symptoms. Older reports suggested only 50% of individuals survived beyond the age of five [1], however a more recent study suggested that about 75% of individuals live to this age [2]. In the vast majority of cases, it has been reported that death occurs secondary to respiratory issues [1].

Mutations in five genes associated with plasmalogen biosynthesis have been reported to underlie RCDP pathology. RCDP1 is the most common type of RCDP and is caused by mutations in the *PEX7* gene, which encodes the PEX7 receptor, responsible for importing alkylglycerone phosphate synthase (AGPS) into the peroxisome [3–7]. The remainder are caused by mutations in one of three genes encoding the peroxisomally active enzymes that perform the initial steps in the plasmalogen biosynthesis pathway: glycerophosphate-O-acyltransferase (*GNPAT*, RCDP2), *AGPS* (RCDP3), and fatty alcohol reductase 1 (*FAR1*, RCDP4) [8–10]. Recently, a specific mutation in the long isoform of *PEX5* was reported in two families and classified as RCDP5. This mutation resulted in impaired binding of PEX5 to the PEX7 receptor, causing problems with peroxisomal targeting [11]. In all cases, the result of these mutations is a severely reduced capacity to synthesize plasmalogens. In fact, while there is no reported correlation between RCDP type and phenotypic severity, there is a direct correlation between phenotypic severity and residual plasmalogen levels in circulation [2, 6, 12–14].

Despite knowledge of the genetic causes of RCDP, there is very little information about the prevalence of the disease. The commonly accepted prevalence value is based on a single, frequently referenced publication, which estimated it at less than 1 per 100,000 births. This study used a population-based register of infants born over an eight-year period in a region of France to evaluate the prevalence of congenital anomalies, finding a single individual out of the 105,374 births examined [15]. In order to appreciate the unmet medical need in the RCDP community, which will drive research into disease management and treatment, it is important to develop a clearer understanding of the true incidence and prevalence of this disease.

Classical epidemiology approaches for determining disease prevalence or incidence are challenging for ultra-rare diseases, as sufficiently large surveys are difficult to conduct, and lack of diagnosis hampers accurate representation. However, since the advent of large-scale genomics studies of general populations, more accurate estimates can be calculated for recessive disorders, using minor allelic frequencies for disease-causing mutations where numerous unaffected carriers are detected [16, 17]. These approaches have already been applied to estimate the disease burden in other rare diseases such as epidermolysis bullosa [18] and autosomal recessive inherited retinal diseases [19].

The goal of this work was to use genetic epidemiology approaches to provide an estimate of the total number of RCDP patients affected and their demographic characteristics. As this model is independent of diagnosis, we expect it to aid in better understanding the unmet medical need of this patient group.

## Results

**Fig. 1:**
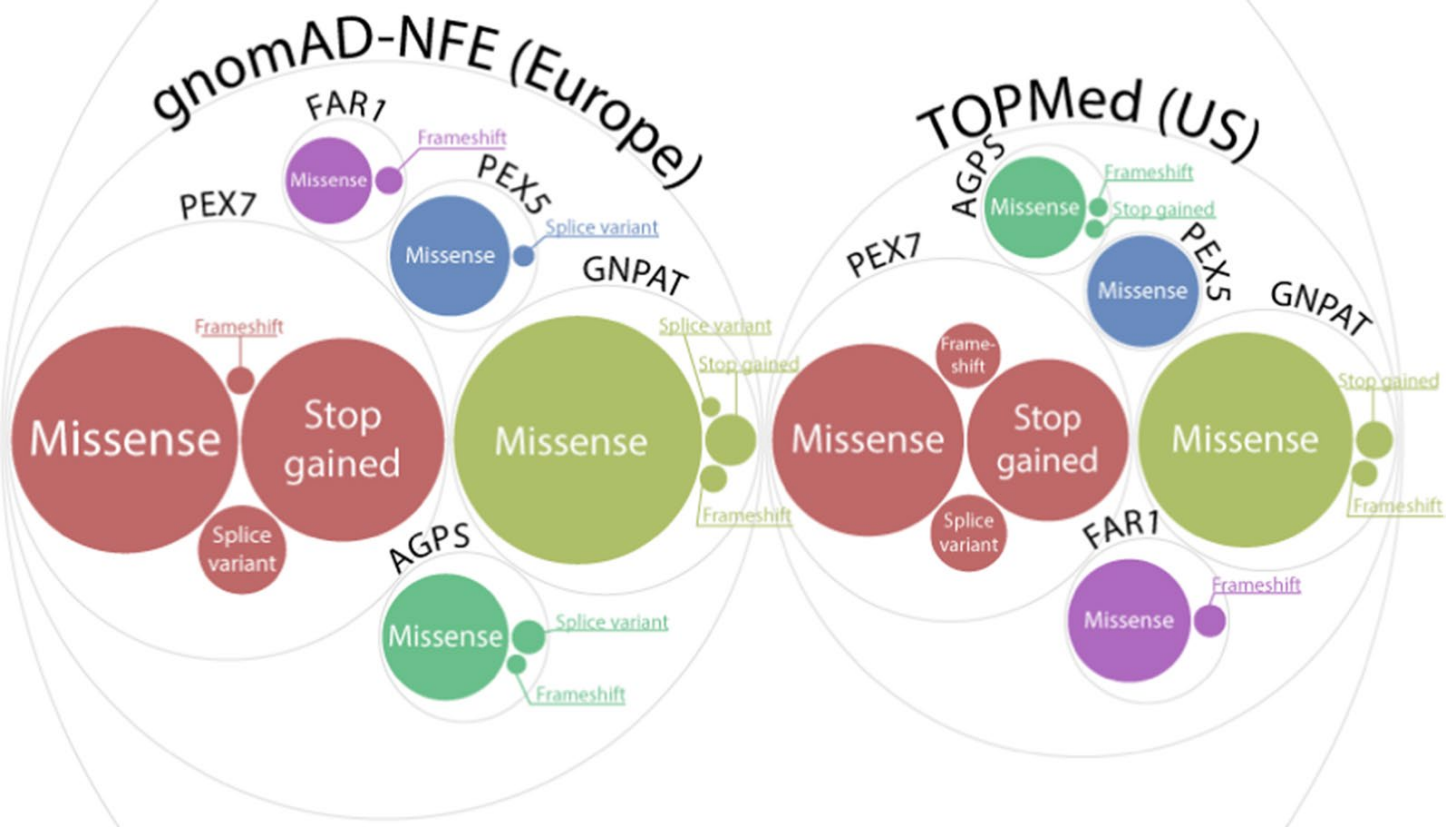
**Overview of the mutational landscape for genes involved in RCDP.** Depiction of variants known or predicted to disrupt protein function for *PEX7, GNPAT, AGPS, FAR1* and *PEX5.* Ball size is proportional to the aggregate allelic frequency for variants identified within the gnomad, non-Finnish European and TOPmed datasets, and clustered according to gene and expected molecular consequence

Review of reported variants in genes: *PEX7*, *GNPAT*, *AGPS*, *FAR1*, and *PEX5* (limited to the region encoding the PTS2 domain) in both TopMed and the gnomAD non-Finnish European (NFE) cohort revealed numerous carriers for clinically reported pathogenic variants associated with RCDP. In addition, in silico evaluation indicated additional variants that we suspect to incur loss of protein function or impair activity. Many of these variants are particularly rare, and may only be described in these large-scale genomics studies. Still, five of the top ten variants contributing to the RCDP1 population in both the US and European cohorts have been previously reported in RCDP cases. Additionally, several more have been reported in ClinVar but require more information to determine the variants’ actual contribution to disease. The full list of variants incorporated into our model can be found in the Additional file 1. Figure 1 represents frequencies of mutations as clustered by molecular consequence contributing to RCDP in each gene and their proportions in the regional datasets. Notably, half of the *PEX7* mutational landscape consists of severe, truncating mutations, in both populations.

While there is a large overlap of mutations present in each dataset, their frequencies vary, therefore contributions to the total population shifts, and some are noticeably missing (Fig. 1 and Additional file 1). For example, five of the top ten most frequent mutations in *PEX7* are shared in the top ten in both datasets, and all mutations in the top ten of the gnomad-NFE cohort are also detected in Topmed. This is not surprising since European ancestry contributes to a large portion of the United States population. However, three of the top ten *PEX7* mutations in Topmed are not detected in gnomad-NFE, but are detected in gnomad cohorts of African and/or Latino genetic ancestry. One of these is the previously reported pathogenic mutation, (rs61753245) p.Trp206Ter [20]. The other two have not been described in the literature but have been reported in ClinVar with uncertain significance (see Additional file 1).

Also noticeably missing from the US Topmed cohort is the *PEX5* splice variant, c.643-2A > C, rs1324287607 (Fig. 1). This is the only reported variant responsible for RCDP type 5 [11], which was described in two separate families with genetic ancestry of the Indian subcontinent. While this *PEX5* splice variant is detected in the gnomad-NFE cohort, there is insufficient data to suggest this variant is enriched in a specific population.

**Fig. 2:**
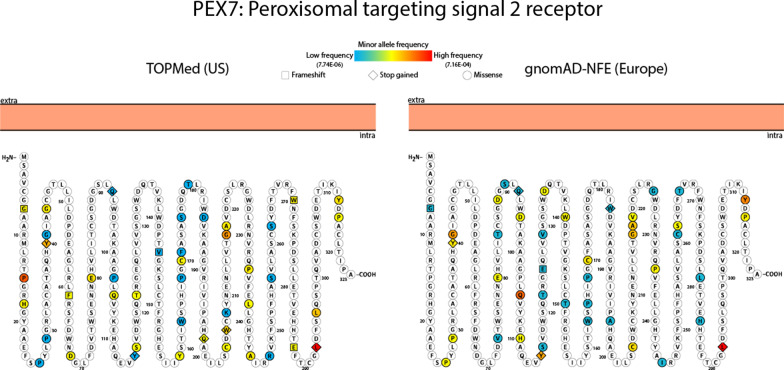
**Coding variants affecting PEX7 identified in TOPmed and gnomad, non-Finnish European datasets.** Known and predicted pathogenic variants were identified in both TOPmed and gnomad-NFE datasets. The expected impact to amino acid sequence is denoted by (□) frameshift, (◊) stop gained and (○) missense. The color scheme indicates the allele frequency found in the respective dataset

Consistent with previous findings in RCDP patients [21], the most frequent variant in all datasets was c.875T > A mutation (rs1805137) in *PEX7*, leading to a premature stop at leucine 292 (Fig. 2 and Additional file 1). Patients carrying this mutation, particularly those homozygous for this variant, present with the classic RCDP phenotype which is associated with extremely low plasmalogen levels [21].

**Fig. 3:**
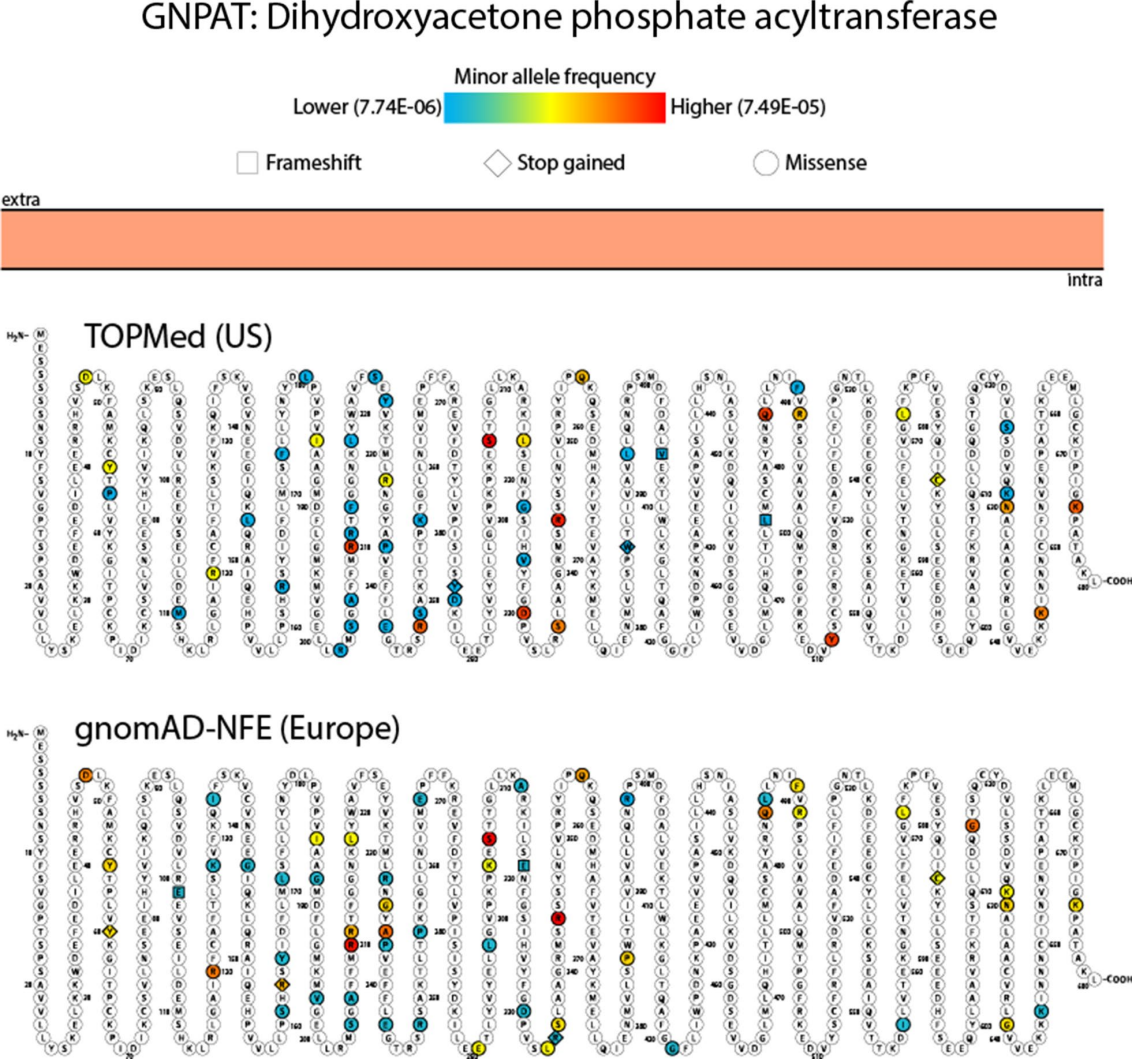
**Coding variants affecting GNPAT identified in TOPmed and gnomad, non-Finnish European datasets**. Known and predicted pathogenic variants were identified in both TOPmed and gnomad-NFE datasets. The expected impact to amino acid sequence is denoted by (□) frameshift, (◊) stop gained and (○) missense. The color scheme indicates the allele frequency found in the respective dataset

Similarly, we observe substantial overlap in the variants identified in *GNPAT* between the two datasets (Fig. 3). While less is known about *GNPAT* contributions to RCDP2 as fewer patients have been identified compared to RCDP1, we predict numerous rare missense mutations may impair GNPAT function. Notably, the two most relatively frequent variants identified in the gnomad-NFE dataset, (rs150822308, p.S206C and rs375611364, p.R344Q) are expected to contribute to approximately one third of GNPAT driven RCDP2 cases. However, neither has been reported in the literature and only rs375611364, p.R344Q has been noted in ClinVar as a variant with uncertain significance (Additional file 1).

### Estimated birth incidence and carriers

The genetic prevalence of pathogenic and carrier genotypes was calculated according to Hardy–Weinberg equilibrium. The genetic prevalence was used to estimate the annual number of births expected to have pathogenic genotypes per gene and population. In addition, the genetic prevalence was used to calculate the total number of estimated carriers of pathogenic mutations according to gene and population. As expected, the most RCDP cases arose from *PEX7* mutations, with an estimated 9–15 children born in the US per year. Similar estimates were observed for Europe, corresponding to the populations of the United Kingdom, Germany, France, Italy and Spain (Table 1). *GNPAT* mutations contribute an estimated additional 4–6 RCDP type 2 children born per year in the US, while children born with RCDP due to AGPS, FAR1 or PEX5 genotypes were expected to be exceedingly rare in both the US and Europe. Nevertheless, the anticipated number of carriers for pathogenic variants in any five genes was estimated to be approximately 2.5 million in the US and 3 million in the largest European countries.

**Table 1:** Estimates of birth incidence for RCDP and carriers per gene and population

Gene	US			EU5 (UK, Germany, France, Italy and Spain)		
	Births	95% confidence range	Carriers (in thousands)	Births	95% confidence range	Carriers (in thousands)
*PEX7*	11.8	(9.3–14.6)	1130	14.2	(11.4–17.4)	1395
*GNPAT*	5.0	(3.7–6.4)	732	7.4	(5.7–9.4)	1006
*AGPS*	0.3	(0.1–0.4)	168	0.5	(0.3–0.8)	269
*FAR1*	0.6	(0.3–0.9)	246	0.1	(0.0–0.2)	126
*PEX5* (PTS2 domain)	0.3	(0.2–0.5)	189	0.4	(0.2–0.6)	228
Total	18	(14–23)	2465	23	(18–28)	3024

### RCDP disease prevalence and age distribution

**Fig. 4:**
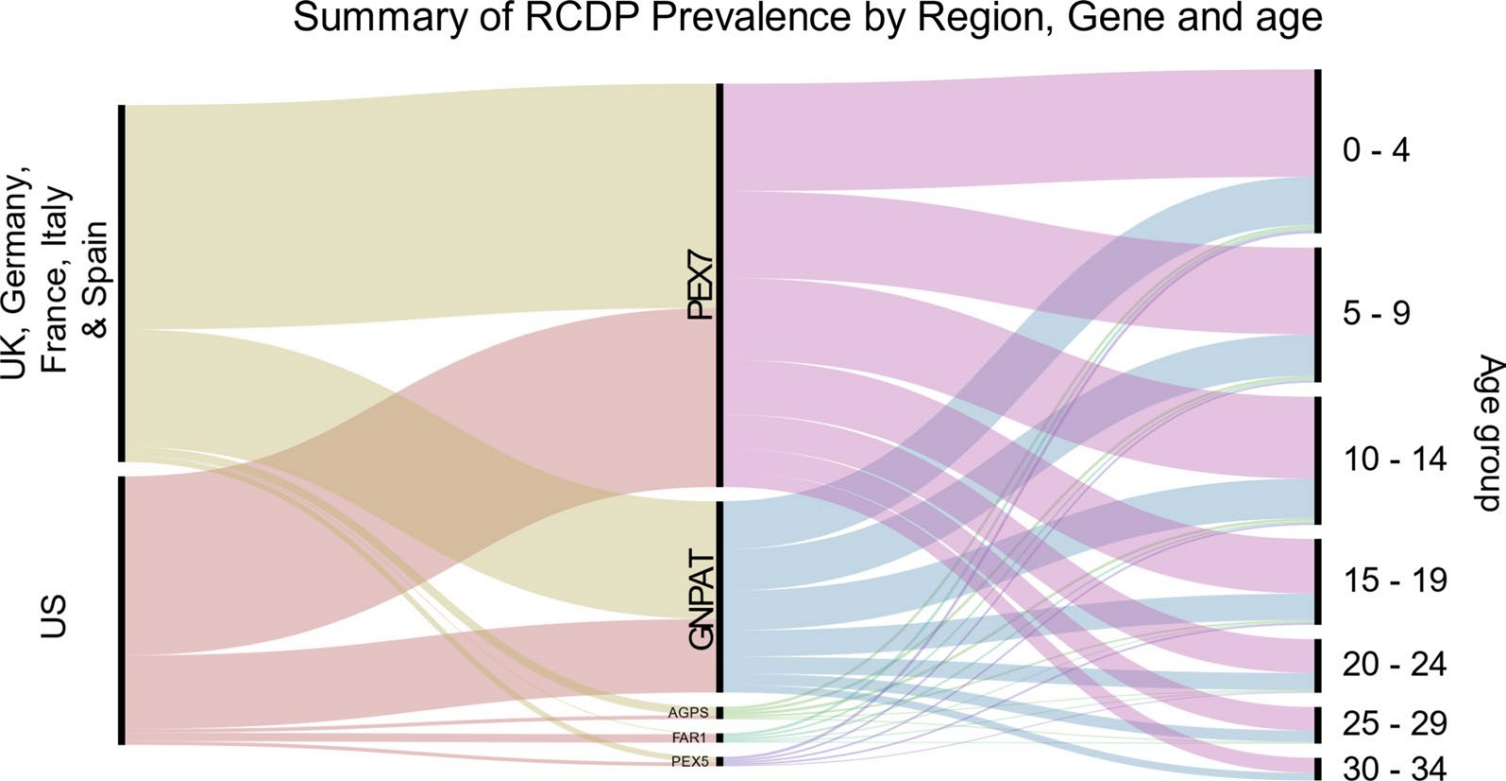
Estimated distribution of RCDP patients by region, gene and age group. This alluvial diagram summarizes the outcome of our genetic based model to describe the estimated prevalence and distribution of RCDP patients in the US and largest European countries

RCDP has a significant impact on life expectancy, and children born with this disease are not expected to reach their third decade of life. To estimate the current disease prevalence of RCDP, we generated a mortality model based on the observations of Duker et al., describing the survival of 66 RCDP patients [2]. Although this report is primarily composed of type 1 RCDP patients, it is the most comprehensive study to date on this patient population, including both US and European patients. We used the combined data of all patients to estimate the mortality rates at each age for all subtypes, and set the maximum lifespan at 34 years old. Based on historic birth data per region and the birth incidence for RCDP children calculated above, we applied our mortality model to estimate the current number of patients per gene and region (Table 2). Our model indicates approximately 190 RCPD type 1 and 80 RCDP type 2 patients may be currently living in the US, with slightly higher projections in the largest European countries. In total, we estimate the whole RCDP patient population in the US is between 219 and 368 patients, and between 297 and 479 patients in the largest European countries. The complete results of our model are summarized in Fig. 4. This includes the age distribution of RCDP patients, with the largest proportion of patients expected to be under the age of 15 years old.

**Table 2:** Current RCDP disease prevalence estimated by gene and population

Gene	US		EU5 (UK, Germany, France, Italy and Spain)	
	Estimated number of patients	95% confidence range	Estimated number of patients	95% confidence range
*PEX7*	190	(150–235)	240	(192–293)
*GNPAT*	80	(59–104)	125	(96–158)
*AGPS*	4	(2–7)	9	(5–14)
*FAR1*	9	(5–14)	2	(1–4)
*PEX5* (PTS2 domain)	5	(3–9)	6	(3–11)
Total	288	(219–368)	382	(297–479)

## Discussion

The model presented here gives a broader picture of the RCDP population in the US and Europe by taking into account the mutational landscape of *PEX7, GNPAT, AGPS, FAR1 * and *PEX5 *(PTS2 domain encoding region) genes on the population level to derive patient estimates in an unbiased manner; independent of diagnosis. Previous estimates of birth prevalence of less than one RCDP case per 100,000 births, at least in Europe [15], is strongly corroborated by our model. We estimated 0.7 and 0.5 RCDP cases per 100,000 births in Europe and the US, respectively. This data further quantitates key trends that have been previously recognized in RCDP clinical research. For example, the clinical recognition that RCDP is most commonly the result of *PEX7* mutations is corroborated by the finding that the *PEX7*, c.875T > A, p.Leu292Ter (rs1805137) mutation has the highest carrier frequency in the US and Europe of all pathogenic and suspected pathogenic variants analyzed and is a commonly identified variant in RCDP patients, including many homozygous [21].

While this model offers one of the most comprehensive views of the RCDP patient population to date, we do consider several limitations that may impact these patient estimates. Firstly, we do assume Hardy–Weinberg equilibriums hold true for this population, which may not always be the case for some specific variants. However, the large sample sizes support the reliability of allelic frequencies. Secondly, we cannot take into account excess fetal mortality that may arise with specific mutations or genotypes that would reduce birth incidence, although such severe mutations would be heavily selected against. Lastly, we considered the carrier rates for genotypes carrying multiple mutations in different target genes to be negligible, given the limited number of variants incorporated into the model. Also, to the best of our knowledge no patients with such complex genotypes have ever been reported.

This model could be further improved with increased ability to predict pathogenic outcomes. It remains challenging to accurately predict certain outcomes like cryptic splicing or other complex regulatory interactions. Although not included in our model, the c.78G > C variant (rs756439083) has been recently implicated in milder RCDP cases, and is indicated to disrupt splicing at the intron 1/exon 1 junction [22]. Using the advanced SpliceAI algorithm [23], based on machine learning, we can predict the original splice donor is likely abolished and a new donor site potentially emerges downstream, which would also impact protein coding (data not shown). Additionally, gene-specific information, such as protein structures would also improve pathogenicity predictions for specific variants.

The biggest impact to this model would be an improved understanding of the genotype–phenotype relationships in RCDP and their impact to life expectancy. Since we have applied the same mortality model for all RCDP subtypes, less severe phenotypes will likely be underestimated. While Duker et al*.* noted there was no significant difference in survival between RCDP type 1 and type 2 patients in their study, they did show that plasmalogen levels could be used to stratify patients as “classic” (severe) and “non-classic” (less severe), resulting in significantly different survival curves between the groups. This is particularly apparent in cases of Adult Refsum’s disease due to *PEX7* mutations, which present with substantially milder symptoms. To date, only three genetically diagnosed patients have been reported in the literature [6, 24, 25], which does not yet provide enough information to relate specific mutations or genotypes to the Adult Refsum disease phenotype. Similarly, persons with *GNPAT* variants leaving residual enzymatic activity may also present with milder RCDP symptoms [14, 26]. These milder phenotypes contributing to the spectrum of RCDP pathogenicity are expected to have relatively longer lifespans but remain largely undiagnosed. As genomics first approaches becomes more mainstream for diagnosing rare disease, the full extent of RCDP phenotypes and any relation to their genotypes will be revealed.

## Conclusions

This study represents the first attempt to estimate the incidence and prevalence of RCDP in the US and Europe. The ultra-rare nature of this disease had previously made accurate assessments of disease prevalence extremely challenging. The availability of large-scale genomic databases has allowed us to use a genetic epidemiological approach to compute the genetic prevalence of RCDP mutations, followed by using modeling of life expectancy to estimate disease prevalence. Our models suggest that there are between 516 and 847 patients in total, currently living in the US and EU5 with RCDP, with another 32–51 born each year. While this still represents a small disease population, it suggests that the unmet medical need of this population is higher than currently appreciated and supports the need for improved diagnostic support, increased disease awareness, and further therapeutic development efforts.

## Methods

Two, non-overlapping, large-scale human genetic diversity datasets were utilized in the construction of the epidemiological models for RCDP; Topmed for representation of the US population [27, 28] and gnomAD_v2 [29], non-Finnish European cohort for representation of populations encompassing the United Kingdom, Germany, Italy, France and Spain. Both of these studies provide sufficiently large genomic datasets for which to extract the minor allele frequencies for known and suspected pathogenic mutations that lead to RCDP.

All clinically identified variants were included in our model, including known splice variants contributing to RCDP. In addition, we included all predicted loss of function variants as well as missense variants that were predicted to be deleterious or damaging by both SIFT and Polyphen-2 for the canonical isoforms [30]. The sum of allelic frequencies per gene were utilized to compute the genetic prevalence of carrier genotypes and pathogenic genotypes according to Hardy–Weinberg distributions. 95% confidence range estimates of allele frequencies were estimated by the modified Wald method [31], which were then applied throughout the model to calculate the upper and lower range estimates. Genotype prevalence was applied to determine the proportion of births in 2018 with RCDP causing genotypes to estimate the birth incidence per gene and region. Historic birth data for specified regions was accessed from the US Federal Reserve Economic data [32] and Eurostat [33].

Based on the data presented by Duker et al. we constructed our mortality model using the determined annual mortality rate per year of life until age 14 and applied a uniform mortality rate of 8% from age 15 onwards. We calculated the historic birth incidence from as far back as 1970 and applied our mortality model using life table analysis to convert incidence to prevalence, by ageing all births to the present. This process was repeated with the lower and upper genetic prevalence values to provide the 95% confidence ranges of the estimates.

## Supplementary Information

**Additional file 1**. Listing of variants per gene included in this model.

## Data Availability

The variant level data from Topmed and gnomad are publicly available can both be accessed from https://www.ensembl.org/index.html.

## Abbreviations

AGPS: Alkylglycerone phosphate synthase
FAR1: Fatty alcohol reductase 1
GNPAT: Glycerophosphate-O-acyltransferase
NFE: Non-Finnish European
RCDP: Rhizomelic chondrodysplasia punctata
